# The effect of WhatsApp-based reminders on enhancing knowledge and adherence to weekly iron-folic acid supplementation among adolescent girls in Maluku, Indonesia

**DOI:** 10.3389/fdgth.2025.1542006

**Published:** 2025-03-27

**Authors:** Mega Clarita Laurence, Christiana Rialine Titaley, Ritha Tahitu, Elpira Asmin, Nathalie Elischeva Kailola, Sean Semuel Istia, Yudhie Djuhastidar Tando, Lershito Antonio Pasamba, Liyani Sartika Sara

**Affiliations:** ^1^ Faculty of Medicine, Pattimura University, Ambon, Indonesia; ^2^ Department of Public Health, Faculty of Medicine, Pattimura University, Ambon, Indonesia

**Keywords:** digital intervention, m-Health, anemia, iron tablet, compliance, female students

## Abstract

**Introduction:**

Anemia continues to be a problem among adolescent girls, including in Indonesia. Although the Weekly Iron-Folic Acid Supplementation (WIFAS) program was introduced in 2014, adherence remains a challenge. The study aimed to evaluate the effectiveness of WhatsApp (WA) reminder messages in improving knowledge and adherence to WIFAS among adolescent girls in the Salahutu Sub-District, Maluku Province.

**Methods:**

A quasi-experimental design was employed in 2024, utilizing a pretest-posttest control group framework across two senior high schools in Salahutu Sub-District. The intervention school (*n* = 49) received WA-based reminder messages for four weeks, while the control school (*n* = 42) continued to receive routine services. We used Mann–Whitney, Fisher Exact and chi-square tests in this analysis.

**Results:**

The WA-based intervention led to a significant improvement in knowledge scores among adolescent girls from the intervention school (*p* *<* *0.001*). These students were also more likely to have taken WIFAS in the week preceding the endline survey (*p* *<* *0.001*) and to have consumed at least 75% of the distributed WIFAS (*p* *=* *0.015*) compared to the control school. Furthermore, the mean hemoglobin levels were significantly higher in the intervention compared to the control school (*p* *=* *0.001*).

**Conclusions:**

The WA-based reminder messages were effective in enhancing knowledge and adherence to WIFAS. Expanding this approach to a broader population is recommended before scaling up implementation across Maluku and other regions in Indonesia.

## Introduction

Anemia in adolescents poses significant health risks, including impaired cognitive function, diminished physical capacity, and, in future pregnancies, complications affecting both maternal and infant health outcomes (1). One of the key interventions implemented by the Government of Indonesia to reduce anemia in adolescent girls is the Weekly Iron-Folic Acid Supplementation (WIFAS) program (1, 2).

The WIFAS program for adolescent girls in Indonesia started in 2014 and has become one of the specific interventions that aim to reduce stunting in children. The Ministry of Health of the Republic of Indonesia recommends all adolescent girls consume iron/folic acid tablets four times monthly or once per week (3). The iron/folic acid tablet contains 60 mg of elemental iron (equivalent to 300 mg ferrous sulfate, 180 mg ferrous fumarate, or 500 mg ferrous gluconate) and 2.8 mg folic acid (a synthetic form of folate).

Despite the program's importance, low adherence to WIFAS has been documented (4–8). According to the 2018 Basic Health Research, 98.6% of adolescent girls who received WIFAS at school did not consume it, as recommended (9). In Maluku Province, one of the largest archipelago provinces in Indonesia, only 0.46% of adolescent girls aged 16–18 years consumed the recommended 52 iron/folic acid tablets obtained from school (10). According to a survey conducted in 2022 by the Faculty of Medicine, Pattimura University, in Ambon, Maluku Province in three senior high schools, only 15% of female students adhered to taking WIFAS and almost 50% of female students were anemic (11). This evidence highlights a strong need for interventions aimed at improving adolescent girls’ adherence to WIFAS especially as existing school-based distribution strategies have not effectively improved compliance, particularly in archipelagic regions like Maluku where access to healthcare services is limited.

Previous literature indicated that digital interventions, including the use of the WhatsApp (WA) application, are part of the effective tools for improving adherence in various health programs (12–15). Studies also reported that the WA application was considered one of the most widely used applications and could effectively be used to improve an individual's knowledge, attitudes, and behaviors related to health (12, 16–21). The application offers instant messaging services that could enhance communication between healthcare providers and their clients by facilitating remote consultations, information sharing, and the timely initiation of treatment (22). Furthermore, the ability of the WA application to feature different types of media, including images, videos, and audio, also sets it apart from other digital health tools that could only use text messages (19). This shows that WA could be considered a valuable tool for making information more attention-grabbing (14). Digital health interventions have been widely studied in urban, well-connected regions, however they were rarely examined in rural and archipelagic settings, where internet penetration, digital literacy, and health behaviors differ.

In early 2024, a survey among senior high school students in the Salahutu Sub-District of Central Maluku District, Maluku Province, was conducted to assess smartphone penetration, internet access, and social media preferences (data not published). The survey revealed that 94% of the 186 respondents owned smartphones, had adequate internet access, and primarily used WA applications. Thereby, based on these findings, in early 2024, researchers from the Faculty of Medicine, Pattimura University, developed some WA-based reminder messages for adolescent girls related to anemia and its prevention as well as reminders to take WIFAS provided at schools. This present study aimed to evaluate the impact of these WA-based reminder messages on knowledge and awareness about anemia and adherence to WIFAS among adolescent girls in Salahutu Sub-District, Maluku Province, Indonesia.

## Materials and methods

### Research design and study site

This quasi-experimental study was conducted from May to June 2024 using a pretest-posttest control group design involving two senior high schools in Salahutu Sub-district of Central Maluku District in Maluku Province, Indonesia. The schools involved were: SMAN 3 Maluku Tengah (intervention school) and SMAN 47 Maluku Tengah (control school).

### Population and sampling

The senior high schools were selected using purposive sampling based on the following criteria: (1) located in Salahutu Sub-District of Central Maluku District; (2) government-owned school; (3) had a minimum enrollment of 100 female students (grades X to XII); (4) received WIFAS from local community health centers; (5) sufficient distance from each other to minimize the risk of contamination; and (5) willingness to participate in the study. Of the two senior high schools selected, one school was randomly selected to become the intervention school.

**Figure 1:**
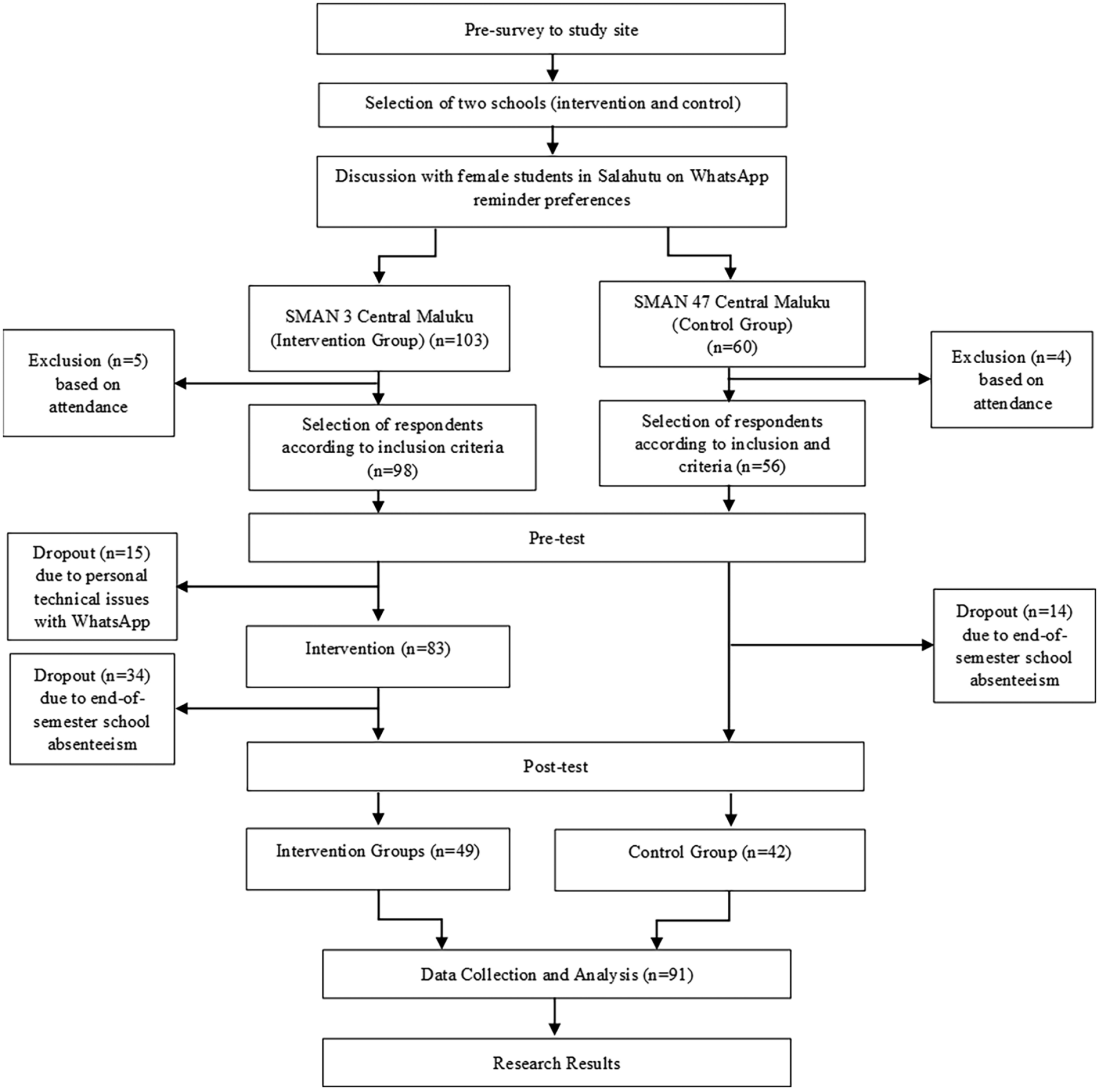
Study participant flowchart.

The inclusion criteria for the study subjects in each high school were: (1) female students from Grades X and XI, and (2) willing to participate in the study. For the intervention school, additional criteria included were: (1) owning a communication device (smartphone, tablet, or laptop) with regular internet access, and (2) having or being willing to install the WhatsApp (WA) application. In total, there were 49 adolescent girls from SMAN 3 Maluku Tengah (intervention school) and 42 from SMAN 47 Maluku Tengah (control school) were included in this study (Figure 1).

### Intervention

The WA-based educational messages, including reminders to take WIFAS, were developed by the research team from the Faculty of Medicine, Pattimura University in Ambon. Initially, a focus group discussion was held with adolescent girls from the Salahutu Sub-District, to gather feedback about the messages developed. This included their preference about the type of message (text, audio, or video), the clarity of the information, as well as other features such as the color preference that could attract their attention. The findings from this discussion were further used by the research team to refine and adjust the WA-based messages.

**Figure 2:**
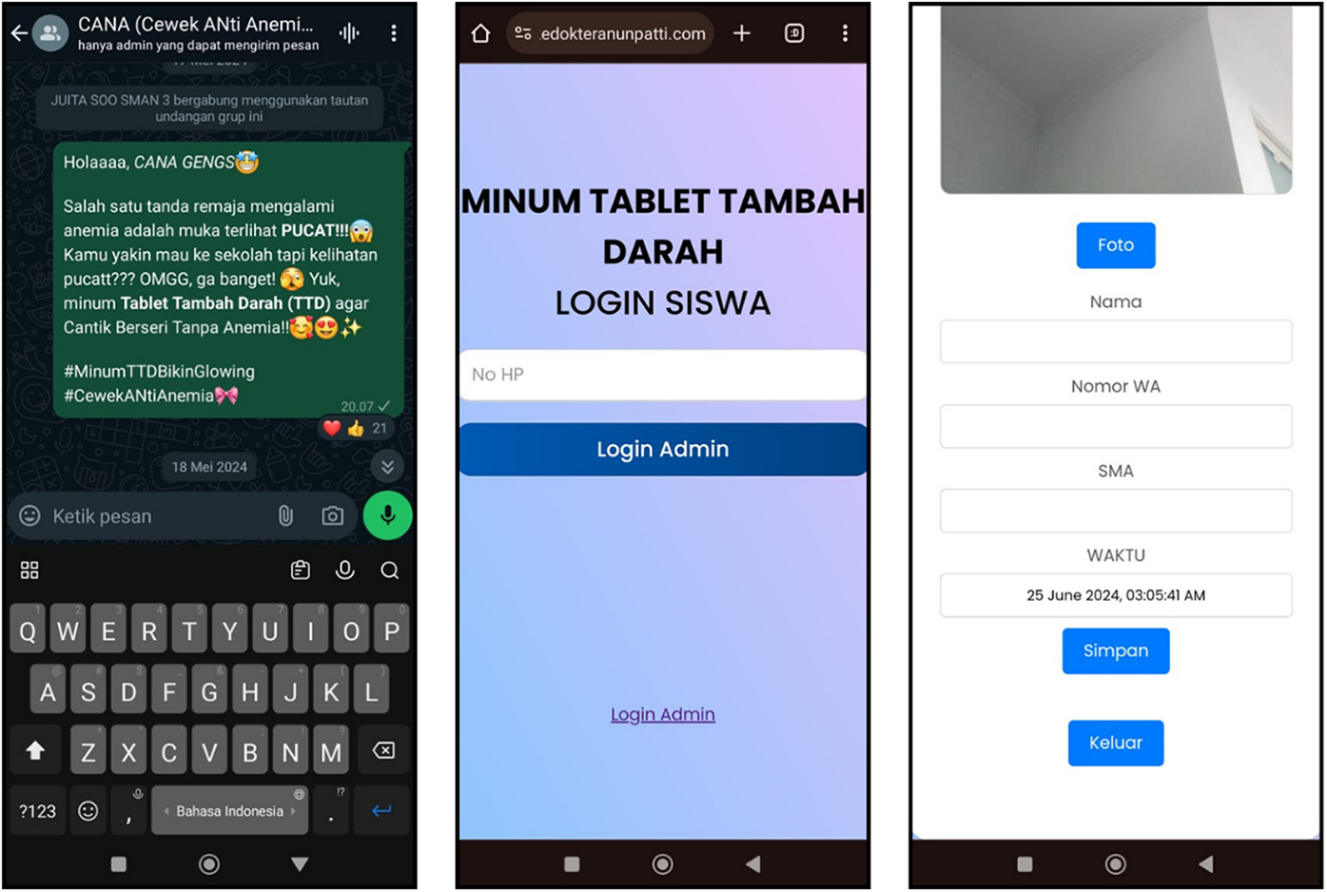
Intervention display and the report system for WIFAS.

The study was conducted for four weeks. In intervention school, in addition to the routine services provided by the local community health center, subjects received WA-based educational messages about anemia and WIFAS, including reminders to take iron-folic acid tablets. These messages, delivered in both text and image formats, were sent to the WA group with all enrolled subjects, three times a week. The reminder messages also included a link for monitoring adherence to WIFAS intake, directing students to a website where they could log in to their accounts. Upon logging in, participants could view their details, and local time, and use a camera feature to record their WIFAS intake for the week (Figure 2).

Students from the control school did not receive any WA-based educational messages and only received routine services provided by local community health centers.

### Evaluation method

The evaluation involved two cross-sectional surveys: before (baseline) and after the intervention (endline). In each survey, respondents were interviewed using a structured questionnaire, and their blood hemoglobin (Hb) levels were measured. After the interviews, all participants received snacks (biscuits and milk).

### Research instrument

This study used a structured questionnaire adapted from a previous study in Indonesia that had been tested for validity and reliability (23). The questionnaire included questions on socio-demographic characteristics, menstrual duration, adherence to WIFAS, and knowledge about anemia and WIFAS. An additional monitoring tool was also used for the intervention school: a link sent with the reminder messages, which connected to a reporting system that tracked the amount of WIFAS consumed by the respondents.

### Variables

The independent variable in this study was the use of WA-based reminder messages containing information about anemia and WIFAS. The dependent variables were knowledge and awareness about anemia and its prevention and adherence to WIFAS. Adherence was defined as the consumption of at least 75% of the WIFAS tablets (three out of four tablets provided).

Knowledge and awareness were assessed using a set of 15 questions designed to measure the respondents’ understanding of anemia and WIFAS, including topics such as anemia signs and symptoms, WIFAS benefits, consumption guidelines, and potential side effects. Each correct answer was scored as 1, and incorrect answers were scored as 0. Knowledge levels were categorized as high or low based on the median score distribution. Scores above the median distribution were considered as high and those below were considered as low.

To confirm adolescent girls’ adherence to taking WIFAS, we measured the Hb level of respondents and the proportion of anemia (Hb < 12 g/dl) among adolescent girls in intervention and control schools. The Hb levels were measured using the HemoCue HB 301 device.

### Data analysis

A descriptive analysis was first conducted to examine the frequency distribution of each variable for both the intervention and control schools at baseline. Using baseline data, the Kolmogorov–Smirnov normality test was applied to assess the distribution of two continuous variables: knowledge about anemia and blood hemoglobin (Hb) concentration. As both variables were not normally distributed (*p* < 0.05), Mann–Whitney tests were used to compare the knowledge scores on anemia and blood Hb concentrations between the control and intervention schools. Additionally, chi-square tests were used to examine differences in self-reported adherence to weekly iron-folic acid supplementation (WIFAS) and anemia status between the two schools at baseline. When the assumptions for the chi-square test were violated, Fisher's exact test was applied.

At the endline, another normality test was conducted to evaluate the distribution of knowledge scores on anemia and blood Hb concentration post-intervention. Since both variables remained non-normally distributed (*p* < 0.05), Mann–Whitney tests were again used to compare differences between the groups. Chi-square tests were also conducted to compare WIFAS adherence and anemia status between the intervention and control schools using endline data. Data analysis was performed using IBM SPSS Statistics for Windows, version 23.

### Ethical approval and informed consent

The study protected respondent privacy by removing personal identifiers, assigning unique codes, and limiting data access to authorized researchers. Electronic data were encrypted, and physical records securely stored, following ethical and institutional privacy standards. The research was approved by the FK UNPATTI Ethics Commission, as confirmed by letter number 014/FK-KOM.ETIK/VIII/2024. Before carrying out the research, the purpose of the study was explained to the respondents, and written informed consent was obtained. In the intervention school, upon agreeing to participate, respondents were asked to join a WA group where educational messages, including reminders to take WIFAS, would be sent.

## Results

In total, we analyzed information collected from 91 students (42 from the control school and 49 from the intervention school). Table 1 presents the sociodemographic characteristics of all respondents involved in this study. In the intervention school, those aged 16 years constituted the highest percentage of respondents and most lived in Negeri Suli (53.1%). Approximately 16% of respondents’ fathers worked in the Indonesian National Armed Forces/Indonesian National Police (*Tentara Nasional Indonesia/Kepolisian Republik Indonesia* or TNI/POLRI) while 73.5% of their mothers were housewives. The average education level of both parents was senior high school (father: 51%; mother: 40.8%).

**Table 1:** Sociodemographic characteristics of adolescent girls in the intervention school.

No	Variable	*n*	%
1.	Age (years)		
	14	1	2,0
	15	15	30.6
	16	19	38.8
	17	13	26.5
	18	1	2.0
2.	Place of residence		
	Liang	1	2.0
	Suli	26	53.1
	Tengah-tengah	2	4.1
	Tial	8	16.3
	Tulehu	9	18.4
	Waai	3	6.1
3.	Father's occupation		
	BUMN	1	2.0
	Labor	3	6.1
	Kuli	1	2.0
	Fisherman	7	14.3
	Farmer	7	14.3
	Civil servants	3	6.1
	Driver	1	2.0
	TNI/POLRI	8	16.3
	Motorcycle taxi driver	3	6.1
	Self-employed	3	6.1
	Don't know	5	10.2
	Died	7	14.3
4.	Father's latest education		
	Finished elementary school	4	8.2
	Finished junior high school	3	6.1
	Finished senior high school	25	51.0
	Graduated from college	1	2.0
	Don't know	16	32.7
5.	Mother's occupation		
	Teacher	6	12.2
	Housewife	36	73.5
	Trader	1	2.0
	Health workers	1	2.0
	Civil servants	1	2.0
	Retire	1	2.0
	Don't know	1	2.0
	Died	2	4.1
6.	Mother's latest education		
	Finished elementary school	5	10.2
	Finished junior high school	5	10.2
	Finished senior high school	20	40.8
	Graduated from college	7	14.3
	Don't know	12	24.5

In this study, we compared the knowledge difference between respondents in the intervention and control schools before and after the intervention, as shown in Table 2. The baseline survey showed that there was no significant difference in the scores of knowledge on anemia and WIFAS between adolescent girls in the intervention and control school (*p* *=* *0.331*). The median knowledge score was 10 for both schools. However, at the endline, a significant difference was found between these two schools, with moderate negative correlation as increased scores were more associated with the intervention group than the control group (r = 0.38 *p* *<* *0.001*). The median score in the intervention school increased to 11, while the median score in the control school remained unchanged (Table 2).

**Table 2:** The median score of knowledge about anemia and weekly iron/folic acid supplementation among adolescent girls in control and intervention schools by the time of the survey.

Time of survey	Group	*N*	Median score	Q1	Q3	*r*	*p*-value*
Baseline	Control	42	10.00	08.00	10.25	0.10	0.331
	Intervention	49	10.00	09.00	11.00		
Endline	Control	42	10.00	08.75	11.25	0.38	<0.001
	Intervention	49	11.00	10.00	12.50		

*Results from Mann–Whitney test.

**Figure 3:**
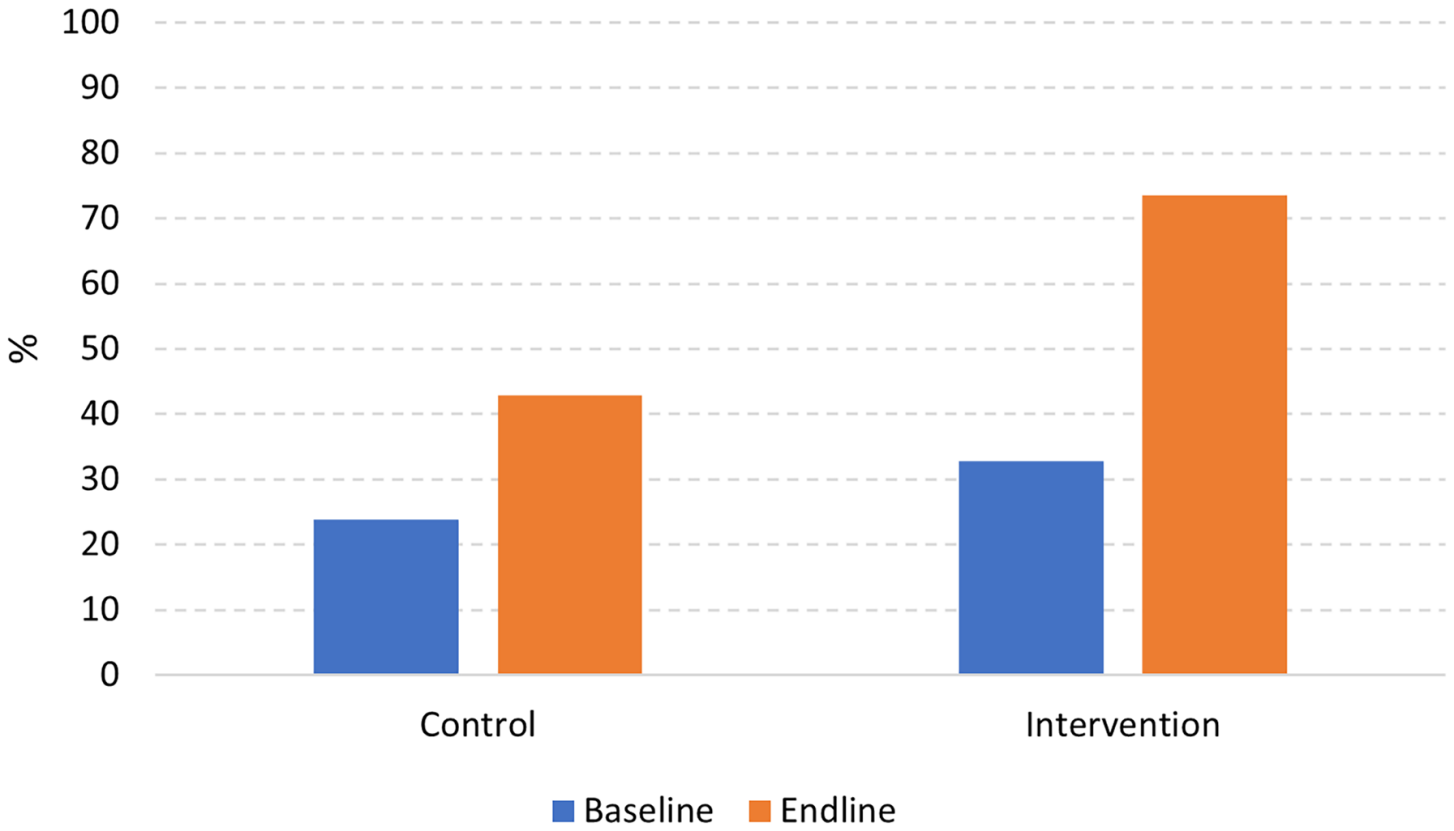
The distribution of respondents with a high level of knowledge about anemia and weekly iron/folic acid supplements among adolescent girls in control and intervention school by the time of the survey.

Figure 3 shows the distribution of respondents with a high level of knowledge about anemia between the control and intervention schools, at baseline and endline. After the intervention, a significant difference in knowledge levels was observed between these two schools (*p* *=* *0.001*). At the endline, 73.5% of respondents demonstrated a high level of knowledge, compared to only 42.9% in the control school.

Table 3 shows the positive effect of WA intervention on adolescent girls’ adherence (consumption of 75% of the iron/folic acid tablets received) to WIFAS. At baseline, there was no significant difference found in the adherence to WIFAS between the two schools (*p* *=* *0.210*). Only 4.8% of respondents in the control and none in the intervention school adhered to WIFAS. After the intervention period, there was a significant difference observed between intervention and control schools (OR = 4.69; 95% CI: 0.06–0.81, *p* *=* *0.025*). Adherence to WIFAS was found in 26.5% of adolescent girls in the intervention school, while only 7% of adherence was found in the control school.

**Table 3:** The level of adherence to weekly iron/folic acid supplementation (consuming 75% of the received tablets) among adolescent girls in control and intervention schools by the time of the survey.

Time of survey	Group	Adherence				Total		OR (95% CI)	*p*-value*
		Adhered		Non-adhered					
		*N*	%	*N*	%	*N*	%		
Baseline	Control	2	4.8	40	95.2	42	100	–	0.210
	Intervention	0	0.0	49	100	49	100		
Endline	Control	3	7.1	39	92.9	42	100	4.69 (1.24–17.83)	0.025
	Intervention	13	26.5	36	73.5	49	100		

*Results from Mann–Whitney test.

The increased consumption of WIFAS among adolescent girls in the intervention school was also mirrored by their increased blood Hb concentration. There was no significant difference in Hb level between the intervention and control school, at baseline (*p* *=* *0.498*) (Table 4). At the endline, the Hb level was significantly higher among subjects in the intervention compared to the control school. There was a moderate positive correlation as increased Hb levels were more associated with the intervention group than the control group (r = 0.35, *p* *=* 0.001). The median Hb level was 13.80 g/dl in the intervention school, and 13.0 g/dl in the control school.

**Table 4:** The median hemoglobin level among adolescent girls in control and intervention schools by the time of the survey.

Time of survey	Group	*N*	Median Hb (g/dl)	Q1	Q3	*r*	*p*-value*
Baseline	Control	42	13.05	12.475	13.675	0.07	0.498
	Intervention	49	12.80	12.150	13.600		
Endline	Control	42	13.00	12.000	13.600	0.35	0.001
	Intervention	49	13.80	12.750	14.300		

*Results from Mann–Whitney test.

In addition to the Hb level, we also assessed the difference in the proportion of anemia between the two schools (Table 5). At baseline, there was no significant difference between the proportion of anemia among adolescent girls in control (19.0%) and intervention school (18.4%) (*p* *=* *0.934*). However, after the intervention, a significant difference was observed, as the control group had 4.5 times higher odds of anemia compared to the intervention group (OR = 4.50, 95% CI: 1.33–15.28, *p* *=* *0.011*). The proportion of anemia in the intervention school was significantly lower in the intervention (8.2%) compared to the control school (28.6%) at endline.

**Table 5:** The proportion of anemia between control and intervention schools by the time of the survey.

Time of survey	Group	Anemia prevalence				Total		OR (95% CI)	*p*-value*
		Anemia		Non-anemia					
		*N*	%	*N*	%	*N*	%		
Baseline	Control	8	19.0	34	81.0	42	100	1.05 (0.36–3.01)	0.934
	Intervention	9	18.4	40	81.6	49	100		
Endline	Control	12	28.6	30	71.4	42	100	4.50 (1.33–15.28)	0.011
	Intervention	4	8.2	45	91.8	49	100		

* Results from chi-square test.

## Discussion

### Main findings

In this analysis, we found that the WA-based reminder messages had a positive effect in enhancing adolescent girls’ knowledge about anemia and their adherence to WIFAS. The endline survey showed significantly higher score of knowledge among respondents in the intervention than control school. Moreover, we also found a significantly higher percentage of adolescent girls who adhered to WIFAS (consuming at least 75% of the tablets received) in the intervention than in the control school. The increased adherence was confirmed by the increased blood Hb concentration and a low proportion of anemia among adolescent girls in the intervention compared to the control school.

The results of this study demonstrate the potential of WA as one of the low-cost digital tools for enhancing health outcomes. These findings suggest that integrating such interventions into school health programs could be an impactful strategy for anemia prevention among adolescent girls in Salahutu Sub-District, Maluku Province.

### The effect of WA-based reminder messages on knowledge of anemia

In this analysis, WA-based messages significantly improved respondents’ knowledge of anemia and WIFAS. This was consistent with findings from previous research (17). Repeated messaging sent through WA helps in reinforcing information that is important to increase adolescents’ awareness and understanding of the health messages (15). As young generations tend to prefer digital platforms, the use of popular applications such as WA could make health messages more engaging. In this study, we attempted to tailor the WA messages based on adolescent girls’ preferences to ensure the content of the messages was engaging, concise, and easy to understand. The regular delivery of visual health information proved to be an effective strategy for educating adolescent girls about anemia, catering to their preference for dynamic learning methods. This was facilitated through the use of the WhatsApp application, which offers a user-friendly interface and a variety of multimedia features, making it an ideal tool for sharing accessible and engaging health information. Consequently, this approach enhanced both the effectiveness and the appeal of health education (24–28).

### The effect of WA-based reminder messages on adherence to WIFAS

We found that WA-based messages significantly improved adherence to WIFAS among adolescent girls. This aligns with previous research demonstrating the effectiveness of digital reminders in enhancing adherence to health interventions (18–20, 26, 28). We assessed adherence by measuring Hb levels in all subjects to confirm their iron and folic acid intake. The intervention school showed significantly higher Hb levels compared to the control school, likely due to improved adherence to WIFAS as reported by the participants from the intervention school. Previous studies have reported similar findings (21). WA-based education has been shown to enhance individuals’ understanding of anemia and its effects on health and quality of life, which may motivate them to take preventive measures more seriously (21, 29, 30). Additionally, the reminder messages sent via WA encouraged participants, especially those prone to forgetting, to adhere to WIFAS. This approach aligns with earlier research indicating that reminder messages can effectively prompt individuals to take specific actions, particularly those who require additional support in remembering or following health recommendations (31). By establishing a routine through repeated health messages, these reminders could reinforce adherence. As the intervention aligns with adolescents’ digital habits, this could make it more acceptable to be integrated into their daily lives. Additionally, the creation of WA groups also fosters a sense of community and promotes peer support. By leveraging the familiarity of WA, these reminders effectively bridge the gap between health recommendations and real-life behavior, which could promote adherence to WIFAS among adolescent girls.

### Policy and research implications

While WA-based reminders improved knowledge and Hb levels, the small effect sizes suggest they may need additional support to drive meaningful change. Stronger engagement strategies, such as interactive discussions, peer support, or incentives, could enhance adherence, while multimedia formats like videos and quizzes may improve knowledge retention. Extending intervention duration and incorporating interactive elements could further strengthen impact. Policymakers should integrate WA-based reminders as a supplementary rather than a standalone strategy, combining digital tools with in-person education to improve WIFAS adherence and reduce anemia. Future research should examine the impact of extended intervention duration, explore alternative engagement methods like gamification or peer-led discussions, and assess the long-term sustainability of adherence. These findings emphasize the potential of digital health interventions within multi-component strategies for lasting behavior change in adolescents.

### Strengths and limitations of the study

This study highlights several strengths associated with the use of WA as a mobile health intervention. First, WA is a widely used, accessible platform that requires minimal technological barriers, making it highly scalable and applicable to diverse populations. Its familiarity ensures ease of use among participants, particularly adolescents, who are accustomed to digital communication. The application is compatible with all smartphone types and includes features such as group chat and broadcasts, which facilitate education and reminders. The WA-based reminder messages were designed based on the results of a focus group discussion conducted with some adolescent girls in the Salahutu Sub-district to ensure that the content was engaging, concise, and easy to understand. Despite these strengths, this study also has some limitations. Internet disruptions, especially during power outages, occasionally delayed message delivery. Although most students had smartphones, varying digital skills among students and teachers may have limited engagement with WhatsApp features. Digital training or alternative platforms could improve accessibility in future interventions. Monitoring adherence was also challenging. While a reporting link was provided, many students did not use it, making real-time tracking difficult. Automated reminders or digital incentives may improve reporting accuracy in future studies. The intervention period was reduced from seven to four weeks due to national holidays, possibly limiting its effectiveness. Additionally, incomplete student participation reduced the sample size, affecting generalizability. Aligning interventions with school schedules and involving teachers or parents could improve participation. These findings highlight the need for better integration of digital tools in school-based supplementation programs. Strengthening technical infrastructure, providing digital training, and incorporating peer or teacher support might enhance engagement and adherence.

## Conclusions

This study demonstrated that WA-based reminder messages effectively improved adolescent girls’ knowledge of anemia and adherence to WIFAS, as indicated by increased Hb levels in the intervention school. These findings highlight the potential of digital health interventions as scalable, cost-effective tools for enhancing adolescent health, particularly in areas with limited healthcare access. Policymakers should integrate WA-based reminders as a supplementary strategy within school health programs, combining digital engagement with in-person education to strengthen WIFAS adherence and reduce anemia prevalence. Schools and community health centers can use familiar digital platforms to deliver engaging health messages and address key barriers like forgetfulness and low motivation. Scaling this approach across Maluku Province and beyond could enhance adolescent anemia prevention efforts. Future research should focus on optimizing intervention duration, testing alternative engagement methods such as gamification and peer-led discussions, and assessing long-term adherence sustainability. Additionally, evaluating cost-effectiveness and integration with existing school-based and healthcare programs is crucial for ensuring broader impact. These findings reinforce the importance of multi-component digital health strategies in driving lasting behavior change and supporting national anemia prevention efforts.

## Data Availability

The raw data supporting the conclusions of this article will be made available by the authors, without undue reservation.
